# Treatment delays for cancer patients in Sub-Saharan Africa: South Africa as a microcosm

**DOI:** 10.3332/ecancer.2024.1747

**Published:** 2024-08-27

**Authors:** Abba Mallum, Saloni Patel, Elizabeth Olatunji, Godwin Nnko, Adewumi Alabi, John Akudugu, Rugengamanzi Eulade, Adedayo Joseph, Mamsau Ngoma, Twalib Athumani Ngoma, Afekhai Taiwo, Maureen Bilinga Tendwa, Mariza Vorster, Wilfred Ngwa

**Affiliations:** 1 School of Clinical Medicine, University of KwaZulu-Natal, Durban 4041, South Africa; 2 Johns Hopkins University School of Medicine, Baltimore, MD 21205, United States; 3 Department of Oncology, Inkosi Albert Luthuli Central Hospital, Durban 4091, South Africa; 4 Muhimbili University of Health and Allied Sciences, Dar es Salaam 17105, Tanzania; 5 Ocean Road Cancer Institute, Dar es Salaam 17105, Tanzania; 6 NSIA-LUTH Cancer Center, Lagos University Teaching Hospital, Lagos 100254, Nigeria; 7 Faculty of Medicine and Health Sciences, University of Stellenbosch, Cape Town 7535, South Africa; 8 Ministry of Health-Rwanda, Kigali, Rwanda; 9 Department of Bioinformatics, Rhodes University, Grahamstown 6139, South Africa; * These authors share first authorship; # Senior author

**Keywords:** Sub-Saharan Africa (SSA), breast cancer, cervical cancer, prostate cancer, time to treatment initiation and treatment delay

## Abstract

**Purpose:**

Delays in initiating cancer treatment time to treatment initiation (TTI) can negatively impact patient outcomes. This study aimed to quantify the association between TTI and survival in breast, cervical and prostate cancer patients at Inkosi Albert Luthuli Central Hospital (IALCH) in KwaZulu-Natal, South Africa, as a microcosm of Sub-Saharan Africa (SSA).

**Methods:**

We analyzed electronic medical records of patients diagnosed with breast, cervical or prostate cancer at IALCH between 2010 and 2020. Median TTI was calculated for different treatment modalities. To assess the link between treatment delay and mortality, we employed a Cox proportional hazards model to estimate hazard ratios (HRs) and 95% confidence intervals (CIs), treating breast cancer and patients over 40 as competing events. Additionally, Kruskal-Wallis one-way analysis and linear regression were used to compare TTI across racial groups.

**Results:**

The study included patients with breast (44%), cervical (44%) and prostate cancer (12%). Mean age at diagnosis was 62.6, 56.6 and 73.0 years, respectively. Breast cancer patients experienced the longest delays for mastectomy (median 18.4 weeks), followed by prostate cancer patients waiting for radiotherapy (median 16.6 weeks). Significantly longer TTI for radiotherapy was observed in patients younger than 40 with cervical (HR = 2.30, 95% CI: 2.16–2.44, *p* < 0.001) or prostate cancer (HR = 1.42, 95% CI: 1.03–1.95, *p* = 0.033) compared to older breast cancer patients. Similar trends were seen for younger patients with cervical cancer receiving chemotherapy. Notably, all racial groups exhibited substantial delays in initiating treatment for all three cancers (breast *p* < 0.001, prostate *p* = 0.004 and cervical cancer *p* < 0.001).

**Conclusion:**

This study identified significant delays in treatment initiation (TTI) for breast, prostate and cervical cancer patients at Inkosi Albert Luthuli Central Hospital (IALCH) in South Africa. These delays were concerning, particularly for younger patients and individuals across all racial backgrounds. Delays in treatment initiation have been linked to increased mortality risk in other studies, highlighting the urgency of addressing this issue. Furthermore, this study serves as a valuable model for future research throughout SSA to collectively address the challenges of treatment delays and improve cancer care for the region.

## Background

Cancer is a formidable global health challenge, with a disproportionate impact on low-and middle-income countries, including those in Sub-Saharan Africa (SSA) [1]. In 2020, SSA reported an estimated 801,392 new cancer cases and 520,158 cancer deaths [2]. Rapid interventions are crucial to mitigate the substantial projected increase in cancer mortality, with data estimates indicating a twofold rise in annual cancer deaths from 2020 to 2040 in this region [1]. Among the most frequent cancers in SSA, breast cancer accounted for 129,415 new cases, cervical cancer for 110,280 new cases and prostate cancer for 77,295 new cases in 2020 [2, 3]. Cervical cancer ranked as the leading cause of cancer-related deaths among women in SSA – exacerbated by HIV and human papillomavirus infections – followed by breast cancer [3]. Prostate cancer was the leading cause of cancer mortality among men [1–4]. Early detection and treatment of cancer are crucial for improving outcomes for patients who undergo radiotherapy, chemotherapy and/or surgical treatment regimens [5]. However, limited access to oncology services in SSA leads to delays in cancer diagnosis and treatment [6].

The impact of treatment delays on survival in various cancer types in SSA is not well understood, with limited estimates hindering comprehensive analysis [7, 8]. Existing studies suggest an association between the time from symptom onset to diagnosis and increased mortality or compromised local control, highlighting the need for interventions that encourage earlier presentation of patients in SSA to oncology centers [9–17]. Delays in cancer care delivery in SSA occur at multiple points along the care continuum, including diagnosis, staging, treatment planning and treatment initiation, and can be categorized as patient-related or system-related [12, 18]. Patient-related delays refer to delays in seeking medical care after symptom discovery, due to factors such as financial challenges and low awareness of cancer symptoms. System-related delays are due to factors in the period between patient presentation at a healthcare facility and therapy initiation, such as low numbers of healthcare facilities and insufficient human resources [2, 19, 20]. Cultural and societal barriers, including cancer-related stigma, also contribute to delays in appointments, diagnostic tests, definitive diagnosis and treatment initiation, at the patient and system level [21–23].

The COVID-19 pandemic highlighted the critical need to understand treatment delays and their impact on cancer patient outcomes [24, 25]. Many SSA countries struggled to provide timely cancer care as resources were diverted to pandemic preparedness, leading to deferred surgeries and treatments [26]. The lack of high-quality data has made it difficult to quantify the impact of these delays [17, 27]. Globally, healthcare systems set guidelines for diagnosis-to-treatment times without substantial empirical support [20]. Addressing this data gap is essential for evidence-based decision-making in cancer care, particularly during pandemics [28]. Understanding the actual impact of treatment delays will help healthcare systems develop effective strategies to ensure prompt treatment and improve patient outcomes in SSA [28].

A recent systematic review and meta-analysis conducted by Hanna *et al* [29] quantified the association between treatment delay and mortality across seven major cancer types and discovered that a 4-week delay in initiating cancer treatment (whether surgical, chemotherapy or radiotherapy) was associated with increased mortality across all cancer types. Due to the lack of robust data analyzing the relationship between treatment delay and survival outcome within SSA countries, there is a paucity of knowledge surrounding the full mortality burden in the region and further research can benefit from applying the results of this analysis to an African population.

This study aimed to expand the current literature by investigating time to treatment initiation (TTI) for breast, cervical and prostate cancer patients at IALCH in Durban, South Africa. Specifically, it sought to quantify TTI and examine its impact on survival outcomes for breast cancer patients, using findings from Hanna *et al* [29]. Additionally, the study analyzed factors, such as race, that may influence TTI, given Durban’s ethnic diversity and documented travel burdens. The ultimate goal was to provide insights into cancer treatment delays in urban SSA and inform future research and policy to improve patient outcomes and address the rising cancer burden in the region.

## Methods

### Ethics approval

The IRB approval number: BREC/00006222/2023 was obtained from Biomedical Research Committee (BREC) of the University of KwaZulu-Natal.

### Study population

#### South Africa

IALCH is a public-private hospital located in Durban, South Africa. The hospital provides surgery, chemotherapy and radiotherapy treatments for cancer patients. Patient information was extracted from the IALCH electronic database for breast, cervical and prostate cancer patients who received initial treatment at the cancer center between January 2010 and December 2020. Collected data included sociodemographic information, date of diagnosis, start date of treatment (including hormonal therapy, surgery, chemotherapy and radiotherapy), and date of death, if applicable. The study encompassed patients diagnosed with stage I, II, III or IV breast, cervical or prostate cancer, in accordance with the guidelines outlined in the American Joint Committee on Cancer, 7th edition [30], during the designated study period.

### Definition of TTI

For the breast cancer analysis at IALCH, TTI was defined as the timeframe spanning from the date of diagnosis to the commencement of treatment for the initial therapeutic intervention, which could involve surgery, chemotherapy, or radiotherapy. For adjuvant indications, such as chemotherapy or radiation administered after surgery, the TTI was defined as the duration between the surgical procedure and the onset of adjuvant treatment. In the case of neoadjuvant treatment administered prior to primary curative therapy, the TTI was determined as the interval between diagnosis and the initiation of neoadjuvant treatment.

Surgery dates were not available for cervical and prostate cancer, thus, TTI for these patient populations was defined as the time from diagnosis to initial treatment with chemotherapy, radiotherapy, hormonal therapy, or chemoradiation, as applicable.

### Statistical analysis

Descriptive statistics were first used to summarize TTI across different treatment intervals, providing an overview of the central tendencies and variability in wait times. To assess the influence of factors such as age, race and cancer type on TTI, we utilized a two-pronged approach. First, a non-parametric Kruskal-Wallis one-way analysis of variance was conducted to identify statistically significant differences in TTI across multiple groups (e.g., age categories, racial groups and cancer types).

Second, for a more detailed analysis of how these factors influence the TTI while accounting for potential confounding variables, a Cox proportional hazards model analysis was performed. This approach estimates the hazard ratio (HR) and its 95% confidence interval (CI) for each factor. The HR indicates the relative risk of experiencing a delayed treatment initiation event (e.g., starting treatment after a certain time) for a specific group compared to a reference group (breast and older patients >40 years of age data). The 95% CI provides a range within which the true HR is likely to fall.

All statistical analyses were conducted using the R software environment, specifically utilizing packages like ‘dplyr’ for data manipulation, ‘lubridate’ for working with dates and times and the ‘survival’ package for survival analysis techniques like the Cox proportional hazards model.

## Results

### Patient characteristics

Between January 2010 and December 2020, IALCH treated more breast (44%) and cervical (44%) cancer patients compared to prostate cancer patients (12%). The mean age of breast cancer patients was 62.6 years, with the majority being of African origin (55%), followed by Asian (30%), Caucasian (9%) and mixed race (3%). Cervical cancer patients had a mean age of 56.6 years, predominantly of African origin (89%), with smaller proportions of Asian (6%), Caucasian (2%) and mixed race (1%) patients. Prostate cancer patients had a mean age of 73.0 years, with most being of African origin (67%), followed by Asian (14%), Caucasian (12%) and mixed race (4%). The remaining percentages in each group had unknown racial origins (Table 1).

### Time to treatment initiation

**Figure 1.**
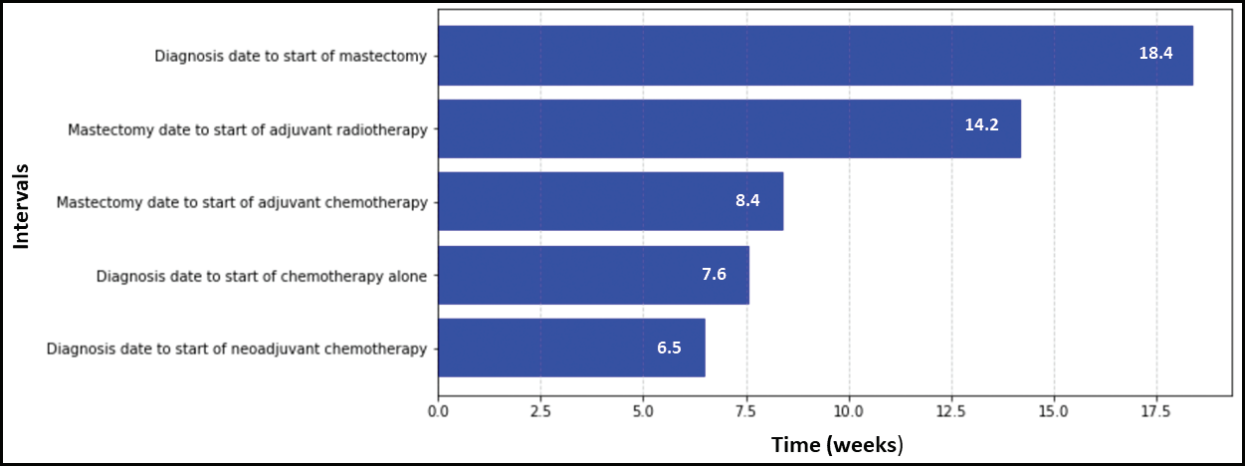
TTI for breast cancer patients at IALCH.

**Figure 2.**
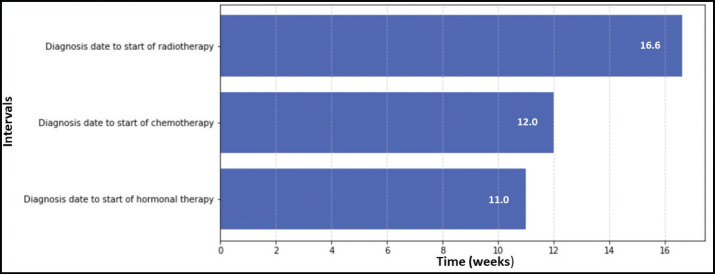
TTI for prostate cancer patients at IALCH.

**Figure 3.**
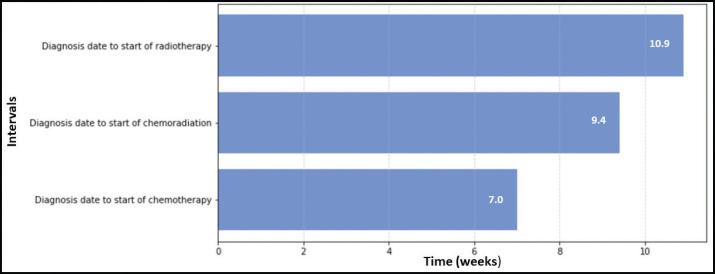
TTI for cervical cancer patients at IALCH.

Figures 1–3 present the median TTI for patients with breast (*n* = 2,569), prostate (*n* = 1,472) and cervical cancer (*n* = 3,322) who received their initial treatment at IALCH. A total of 7,363 patients across these three cancer types were included in the TTI analysis.

Figure 1 specifically focuses on breast cancer patients, revealing variations in TTI based on treatment modality. The waiting time from diagnosis to the start of mastectomy (median: 18.4 weeks) was significantly longer compared to neoadjuvant chemotherapy (median: 6.5 weeks) and radiotherapy (median: 14.2 weeks). Additionally, the figure shows the waiting period for adjuvant chemotherapy (median: 8.4 weeks) following mastectomy was longer than those who received chemotherapy (median: 7.6 weeks) alone.

An analysis of TTI for prostate cancer patients revealed that radiotherapy had the longest median waiting time from diagnosis (16.6 weeks). Conversely, hormonal therapy (median: 11.0 weeks) and chemotherapy (median: 12.0 weeks) had the shortest median waiting times for initiation of treatment (Figure 2).

Figure 3 illustrated the variations in TTI for cervical cancer patients. Radiotherapy had the longest median waiting time from diagnosis (10.9 weeks). Conversely, chemotherapy had the shortest median waiting time (7.0 weeks), followed by chemoradiation (combination of chemotherapy and radiotherapy) at 9.4 weeks.

#### TTI compared by racial category (IALCH)

**Figure 4.**
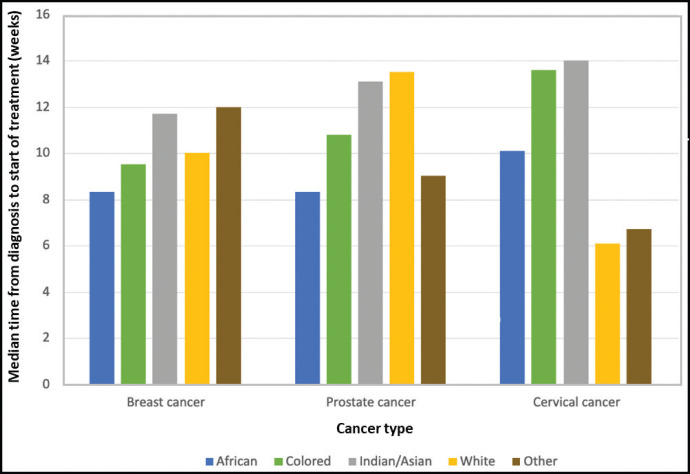
A graphical representation of TTI by race (IALCH) for breast, prostate and cervical cancer.

Table 2 displays the results of the Kruskal-Wallis one-way analysis of variance test comparing TTI by racial category for patients at IALCH. Patients whose race was unknown were excluded from the analysis. A difference was observed for TTI between racial groups for all three cancer types assessed. The results are summarized in Figure 4.

### Estimation of HR

**Figure 5.**
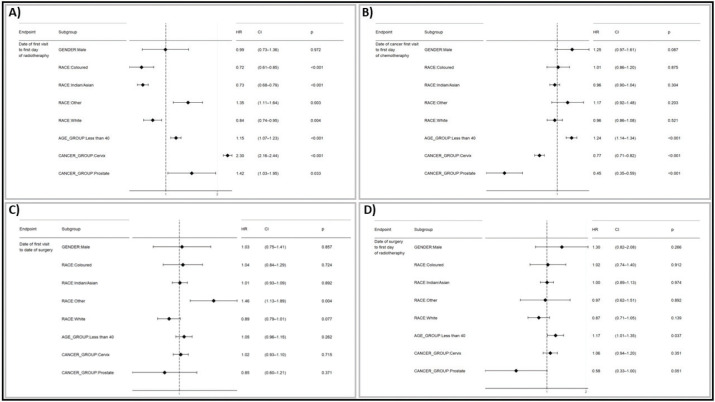
A graphical representation comparing TTI by race (IALCH) and a forest plot visualizing the HR for delays in treatment initiation from the first visit to the first date of (a): Radiotherapy, (b): Chemotherapy, (c): Surgery and (d): Surgery to the first day of radiotherapy. This comparison includes various subgroups using prostate and cervical cancer as references, compared to breast cancer.

The following images depict the HR and 95% CI from a Cox proportional hazards model analysis on a population of breast, cervical and prostate cancers (Figure 5). The analysis was conducted in R using dplyr, lubridate and survival analysis package.

#### Date of first cancer visit to radiotherapy

Figure 5a revealed significant disparities in the time to initiation of radiotherapy across various subgroups. Patients identified as ‘Coloured’ (HR = 0.72, 95% CI: 0.61–0.85, *p* < 0.001), ‘Indians/Asians’ (HR = 0.73, 95% CI: 0.68–0.78, *p* < 0.001) and ‘White’ (HR = 0.84, 95% CI: 0.74–0.95, *p* = 0.004), had significantly shorter times to start radiotherapy compared to reference group (African patients). Conversely, patients in the ‘Other/Unknown’ racial category (HR = 1.35, 95% CI: 1.11–1.64, *p* = 0.003) and those younger than 40 years old (HR = 1.15, 95% CI: 1.07–1.23), *p* < 0.001) experienced longer delays in initiating radiotherapy. Additionally, patients with cervical cancer (HR = 2.30, 95% CI: 2.16–2.44, *p* < 0.001) and prostate cancer (HR = 1.42, 95% CI: 1.03–1.95, *p* = 0.033) had longer times to start radiotherapy compared to breast cancer patients. No significant difference was observed between male and female patients.

#### Date of first cancer visit to chemotherapy

Figure 5b depicts the risk factors associated with initiating chemotherapy treatment. Patients aged less than 40 years (HR 1.23, 95% CI: 1.14–1.33, *p* < 0.001) and those with cervical cancer (HR 0.71, 95% CI: 0.67–0.77, *p* < 0.001) exhibited significantly different risk of transitioning to chemotherapy compared to their respective reference groups (those who are above age 40 and breast cancer). The HR for the subgroups, such as race (Colored, Indian/Asia, Other, White) and prostate cancer who were not statistically significant at the 0.05 levels, as indicated by the CI spanning 1 and the *p*-values exceeding 0.05.

#### Date of first cancer visit to surgery

The HR estimates suggested the race ‘Other/Unknown subgroup’ (HR 1.46, 95% CI 1.13–1.89, *p* = 0.004) exhibited a statistically significant increased risk of having a longer time between surgery and cancer first visit compared to the reference African race group. On the other hand, the HRs for subgroups such as gender (male), race (Colored, Indian/Asian, White), age group (less than 40) and cancer type (cervix, prostate) were not statistically significant, as indicated by CI spanning 1, and *p*-values exceeding the conventional 0.05 level (Figure 5c).

#### Date of surgery to the first day of radiotherapy

Patients with cervical cancer (HR 0.78, 95% CI: 0.69–0.89, *p* < 0.001) had a significantly shorter time between surgery and initiation of radiotherapy. Similarly, patients with prostate cancer also had a significantly shorter time between surgery and radiotherapy initiation (HR 0.42, 95% CI: 0.24–0.78, *p* = 0.002). The HR for other subgroups, such as gender, race and age group (less than 40), were statistically not significant as indicated by CI spanning 1 and the *p*-value exceeding the conventional 0.05 level.

## Discussion

This study investigated TTI for prostate, breast and cervical cancer patients at a large urban hospital in KwaZulu-Natal, South Africa. The findings align with existing data on cancer prevalence in SSA, with breast and cervical cancers being most common among women and prostate cancer leading in men [31, 32]. The median diagnosis age in our study also mirrored broader African trends [33, 34].

However, an important aspect of this research lies in the observed disparities in TTI. Patients younger than 40 with cervical (HR = 2.30, 95% CI: 2.16–2.44, *p* < 0.001) or prostate (HR = 1.42, 95% CI: 1.03–1.95, *p* = 0.033) cancer experienced significantly longer waits for radiotherapy compared to older breast cancer patients. Similar trends were seen for cervical cancer treated with chemotherapy. These findings are concerning, as numerous studies have established a link between delayed treatment initiation and increased mortality risk across various cancers. Studies report a 6%–13% rise in mortality risk with each month of treatment delay for specific cancers and treatment modalities [29, 35]. Delays in treatment for breast and cervical cancer have been shown to significantly worsen survival outcomes [29, 36–39]. While the impact of delayed treatment on prostate cancer mortality remains under investigation, longer TTI can negatively affect treatment compliance and continuity of care, potentially impacting patient outcomes [29, 40–42].

Our study identified racial disparities in TTI, with all racial groups (African, Asian, Coloured, White and others) experiencing significant delays in treatment initiation for breast (*p* < 0.001), prostate (*p* = 0.004) and cervical cancer (*p* < 0.001). However, it is important to acknowledge a limitation of this analysis. Socioeconomic factors, which can vary significantly across racial groups, were not considered. These factors can significantly influence healthcare access and treatment-seeking behavior, potentially confounding the observed association between race and TTI [43]. Future research employing multivariate analysis is crucial to understand the underlying mechanisms and potential confounders contributing to these racial disparities [44]. Existing literature suggests that cultural factors, such as lack of cancer awareness and stigma, can create barriers to seeking diagnosis and treatment, particularly among men and women in India and SSA [21, 45–49]. Similar cultural attitudes could be present within South Africa’s Asian Indian immigrant communities as well as African populations, potentially contributing to the observed racial variations in TTI.

Geographic limitations are another significant barrier to timely treatment in SSA. Limited access to radiotherapy services, particularly in areas with scarce radiotherapy machines, creates delays and negatively impacts patient outcomes [50]. Additionally, lengthy travel distances can hinder treatment compliance due to the demands of radiotherapy schedules and frequent hospital visits [51]. Efforts to increase access to radiotherapy services, such as implementing hypofractionated treatment schedules, could alleviate these burdens for patients across SSA [52, 53].

There are several limitations to this study. First, our analyses are limited to urban centers within SSA, and do not reflect the realities of cancer treatment delays in other parts of each country. In particular, access to cancer care is significantly more limited in rural SSA, and patients within these regions likely experience prolonged treatment delays and worse survival outcomes than reported within our study population [36]. Second, the retrospective approach offers a single point in time for analysis. Longitudinal studies that follow patients over time are needed to definitively establish a link between treatment delays and mortality risk. Additionally, the study design did not account for social determinants of health like poverty, education level and social stigma, which significantly influence healthcare access and treatment-seeking behaviors [54, 55]. Finally, we were limited in our ability to analyze delays in time to surgery for most of our study population, which is an important investigation since surgery services are severely limited across SSA and there remains a significant shortage of oncologic surgeons in the region [37].

Despite these limitations, our study is, to the best of our knowledge, one of the first to estimate the impact of treatment delay on mortality risk for patients in a country within SSA. Future research on this topic can benefit from adopting a longitudinal approach to develop survival models that are unique to African populations and more accurately capture the relationship between treatment delay and mortality risk. Previous studies have calculated treatment delays and examined their causal factors in various SSA countries [38–40], and adapting an Afro-centric survival model to these analyses can help illuminate the true mortality burden associated with prolonged time to cancer treatment. As the incidence of cancer continues to grow throughout SSA, understanding this relationship is an integral component of mitigating the burden of disease.

## Conclusion

Median TTI for breast, prostate and cervical cancer patients attending treatment at a large, urban oncology center in Durban, South Africa, was prolonged and associated with increased mortality risk for delays in breast cancer treatment. There were differences in TTI based on racial category (IALCH), is needed to uncover the underlying sociocultural mechanisms for these findings. As the burden of cancer continues to increase in South Africa and SSA as a whole, addressing delays in treatment initiation may be valuable for decreasing patient mortality in the region.

## Conflicts of interest

The authors declare no conflicts of interest.

## Author contributions

Conceptualization and design: SP, EO, AM, GN, WN. Project administration and supervision: AA, AJ, AT, MN, TAN. Investigation: SP, EO, GN, JA, RE, MBT, MV. Data interpretation: SP, EO, GN, RE, MBT, MV. Writing-original draft: SP, EO, AM. Writing-reviewing and editing: all authors. All authors contributed to the article and approved the submitted version.

## Figures and Tables

**Table 1.** Characteristics of the study population at IALCH.

Study site - IALCH	*N* = 12,657 (%)
Breast cancer Mean age (years) Race African White Colored Asian Indian Other Unknown Prostate cancer Mean age Race African White Colored Asian Indian Other Unknown Cervical cancer Mean age Race African White Colored Asian Indian Other Unknown	5,545 (44) 62.6 3,062 (55) 512 (9) 181 (3) 1,635 (30) 53 (1) 102 (2) 1,553 (12) 73.0 1,045 (67) 185 (12) 69 (4) 221 (14) 8 (1) 25 (2) 5,559 (44) 56.6 4,917 (89) 94 (2) 76 (1) 325 (6) 19 (0) 128 (2)

**Table 2.** TTI compared by race (IALCH).

Cancer type	Race	*n*	Median TTI^a^ (weeks)	IQR^b^	*p*-value
Breast cancer	African	2,298	8.3	16.6	
	Colored	1,253	9.5	27.7	
	Asian Indian	126	11.7	36.3	
	White	356	10	18.7	
	Other	41	12	38.1	
					< 0.001
Prostate cancer	African	632	8.3	18.8	
	Colored	44	10.8	20.1	
	Asian Indian	144	13.1	88.6	
	White	6	13.5	36.4	
	Other	98	9	51.7	
					0.004
Cervical cancer	African	2,967	10.1	15.3	
	Colored	53	13.6	25.3	
	Asian Indian	203	14.0	24.2	
	White	51	6.7	13.0	
	Other	8	6.1	6.3	
					< 0.001

a TTI

b Interquartile range (IQR)
